# ATRX-Deficient High-Grade Glioma Cells Exhibit Increased Sensitivity to RTK and PDGFR Inhibitors

**DOI:** 10.3390/cancers14071790

**Published:** 2022-03-31

**Authors:** David Pladevall-Morera, María Castejón-Griñán, Paula Aguilera, Karina Gaardahl, Andreas Ingham, Jacqueline A. Brosnan-Cashman, Alan K. Meeker, Andres J. Lopez-Contreras

**Affiliations:** 1 Department of Cellular and Molecular Medicine, DNRF Center for Chromosome Stability and Center for Healthy Aging, University of Copenhagen, 2200 Copenhagen, Denmark; davidpm@sund.ku.dk (D.P.-M.); maria.castejon@cabimer.es (M.C.-G.); paula.aguilera@cabimer.es (P.A.); kgaardahl@sund.ku.dk (K.G.); ingham@sund.ku.dk (A.I.); 2 Centro Andaluz de Biología Molecular y Medicina Regenerativa (CABIMER), Consejo Superior de Investigaciones Científicas (CSIC), Universidad de Sevilla, Universidad Pablo de Olavide, 41013 Seville, Spain; 3 Department of Pathology, Johns Hopkins University School of Medicine, Baltimore, MD 21287, USA; jackiebros@gmail.com (J.A.B.-C.); ameeker1@jhmi.edu (A.K.M.)

**Keywords:** glioblastoma, glioma, ATRX, RTKi, PDGFRi, drug screen

## Abstract

**Simple Summary:**

High-grade glioma has a poor prognosis and new effective strategies to treat this aggressive form of cancer are highly needed. We have conducted a drug screen searching for compounds toxic to ATRX-deficient cells, a frequent scenario in cancer, and particularly in high-grade gliomas. We have identified that ATRX-deficient glioma cells are sensitive to several multi-targeted receptor tyrosine kinase and specific platelet-derived growth factor receptor inhibitors, some of which are currently under study in clinical trials. In view of our results, we believe that taking into consideration the presence/absence of ATRX mutations could provide valuable information to interpret the results of those clinical trials.

**Abstract:**

High-grade glioma, including anaplastic astrocytoma and glioblastoma (GBM) patients, have a poor prognosis due to the lack of effective treatments. Therefore, the development of new therapeutic strategies to treat these gliomas is urgently required. Given that high-grade gliomas frequently harbor mutations in the SNF2 family chromatin remodeler *ATRX*, we performed a screen to identify FDA-approved drugs that are toxic to ATRX-deficient cells. Our findings reveal that multi-targeted receptor tyrosine kinase (RTK) and platelet-derived growth factor receptor (PDGFR) inhibitors cause higher cellular toxicity in high-grade glioma ATRX-deficient cells. Furthermore, we demonstrate that a combinatorial treatment of RTKi with temozolomide (TMZ)–the current standard of care treatment for GBM patients–causes pronounced toxicity in ATRX-deficient high-grade glioma cells. Our findings suggest that combinatorial treatments with TMZ and RTKi may increase the therapeutic window of opportunity in patients who suffer high-grade gliomas with *ATRX* mutations. Thus, we recommend incorporating the *ATRX* status into the analyses of clinical trials with RTKi and PDGFRi.

## 1. Introduction

Inherited mutations in the SNF2 family chromatin remodeler *ATRX* cause the alpha thalassemia mental retardation X- linked syndrome, which this protein is named after [1]. *ATRX* is also a bona fide tumor suppressor gene frequently mutated in several cancer types [2]. ATRX together with DAXX (Death domain-associated protein 6) acts as a histone chaperone to deposit the histone variant H3.3 in a replication-independent manner at heterochromatic regions of the genome [3,4]. ATRX is involved in the maintenance of genome stability in cells by several mechanisms. For instance, the deposition of histone H3.3 by ATRX/DAXX is important for timely and accurate double-strand break (DSB) repair by homologous recombination [5]. Furthermore, ATRX suppresses genome instability at telomeres and common fragile sites (CFS), and it has been shown to suppress R-loop formation and promote the resolution of G-quadruplexes [6,7,8]. Thus, loss of ATRX leads to increased genome instability manifested as elevated DSBs, increased CFS expression, and micronuclei formation [5,7,8], which can influence tumor progression and therapy response in cancer patients. *ATRX* mutations are also often associated with the Alternative Lengthening of Telomeres (ALT) in cancers [9], although the loss of *ATRX* alone is not sufficient to trigger the ALT phenotype [9,10]. In addition, ATRX is a critical regulator of therapy-induced senescence, since ATRX-depleted cells are deficient in triggering the cell senescence program [11].

*ATRX* mutations occur in a variety of human cancers such as hepatocellular carcinoma, pancreatic neuroendocrine tumors, and gliomas [12,13,14]. Often, these mutations are truncating mutations that lead to a loss of the functional protein [15]. *ATRX* mutations are associated with *PDGFR* amplification [16,17] and with mutations in the *TP53* and *IDH1* genes in cancer cells. On the contrary, *ATRX* and *DAXX* mutations are mutually exclusive in glioma and other cancers [2,18]. Interestingly, in the rare pancreatic neuroendocrine cancer, *ATRX/DAXX* inactivating mutations are frequently associated with mutations of genes in the mTOR pathway [13,19]. Furthermore, it has been described that inactivating *ATRX* mutations are mutually exclusive with *MYCN* amplification in neuroblastoma [20]. High-grade gliomas often harbor *ATRX* mutations frequently co-occurring with mutations in the *TP53* and *IDH1* genes in this type of malignancy (reviewed in [21]). Glioblastoma Multiforme (GBM) is the most prevalent malignant type of glioma in adults [21]. The overall survival rate of GBM is less than one year from the time of diagnosis. Currently, the first line of treatment for GBM patients, if tumor resection is not possible, is the administration of the DNA alkylating agent temozolomide (TMZ) and radiotherapy, which increases patient survival by an average of 2.5 months [22]. Therefore, the low overall survival rate and the current lack of effective treatments highlight the need for new and more effective drugs for GBM treatment. In line with this, a recent study reports that one promising possibility for targeting ATRX-deficient cancers is the use of WEE1 inhibitors [23]. However, WEE1 inhibitors are not clinically approved.

In this study, we aimed to identify drugs that exhibit synthetic lethality with ATRX loss and, thus, exploit ATRX deficiency for the treatment of high-grade glioma patients harboring *ATRX* mutations. Therefore, we compared the toxicity profiles of ATRX-proficient versus ATRX-deficient cells exposed to a compound library of 1496 FDA-approved drugs. We demonstrate that four multi-targeted receptor tyrosine kinase inhibitors (RTKi) and a specific inhibitor targeting the platelet-derived growth factor receptor (PDGFRi) exhibit higher toxicity in ATRX-deficient high-grade glioma cells. Furthermore, we show that ATRX-deficient cells are highly sensitive to combinatorial treatments of TMZ and RTKi or PDGFRi. The RTKi tested in this study (nintedanib, sunitinib, pazopanib, and sorafenib) are currently being assessed in multiple clinical trials for GBM treatment and other cancer types. Our findings suggest that the status of ATRX should be considered to stratify patients when evaluating the efficiency of RTKi in those clinical trials.

## 2. Methods

### 2.1. Cell Culture

HeLa cells were grown in DMEM (Dulbecco’s modified Eagle’s medium) supplemented with 10% of fetal bovine serum (FBS) (Life Technologies, Rocky Hill, NJ, USA) and 1% of penicillin/streptomycin (Life Technologies). The glioma cell lines MOG-G-UVW (grade III human adult anaplastic astrocytoma) and SF188 (grade IV human pediatric GBM) were grown in DMEM/F12 (DMEM nutrient mixture F-12) and U-251 (grade IV human adult GBM) cells in RPMI (Roswell Park Memorial Institute) medium. DMEM/F12 and RPMI mediums were supplemented with 10% FBS, 1% penicillin/streptomycin, 1% amphotericin B (Sigma, St. Louis, MO, USA), 10 μg/mL gentamicin (Sigma), and 5 μg/mL Plasmocin (Invitrogen, Carlsbad, CA, USA). Patient-derived glioma cells (all grade IV human adult GBM) were grown in Neurobasal and DMEM/F12 glutamax (ratio 1:1) supplemented with 1% of penicillin/streptomycin, N2, B27 (Life Technologies), EGF (10 ng/mL) and bFGF (10 ng/mL) (Peprotech, Rocky Hill, NJ, USA). These cells from the human glioblastoma cell culture (HGCC) collection were grown as adherent monolayers in laminin-coated dishes. All the cell lines were maintained at 37 °C in a 5% CO_2_ incubator.

### 2.2. Generation of ATRX KO Cells

ATRX KO HeLa cells were generated by CRISPR/Cas9 following Ran et al., 2013 protocol [24]. The guide RNA (gRNA) sequences that were used to generate the plasmid were as follows: 5′-CACCGCAGGATCGTCACGATCAAAG-3′ (forward) and 5′-AAACCTTTGATCGTGACGATCCTGC-3′ (reverse). The gRNA was cloned into pSpCas9(BB)-2A-GFP vector (#48138, addgene) for co-expression with Cas9. HeLa cells were seeded in 6-well dishes at a density of 70% a day prior transfection. The cells were transfected with 1.25 µg of the sequence-verified gRNA cloned into pSpCas9(BB)-2A-GFP plasmid using Fugene (Promega, Madison, WI, USA). The day after, GFP-positive single cells were sorted into a 96-well plate using a FACS Aria-II cell sorter. Clonal cell lines were expanded for 2 weeks and screened by Western blot analysis.

### 2.3. Western Blot

Cells were lysed in RIPA buffer (Sigma) supplemented with complete protease inhibitor cocktail tablet (Roche, Basel, Switzerland), 5 mM β-glycerolphosphate (Sigma), 5 mM sodium fluoride (Sigma) and 1 mM sodium orthovanadate (Sigma). Then, lysis extracts were centrifuged at 13,000 rpm for 15 min at 4 °C and supernatants were collected for protein measurement. Protein quantification of the cell lysates was performed with the DC Protein Assay kit (Bio-Rad, Richmond, CA, USA) according to supplier’s instructions. Cell lysates containing 30 μg protein were boiled (4:1 ratio) in NuPAGE™ LDS Sample Buffer 4x (Invitrogen) with 10 mM DTT for 15 min at 70 °C. An equal amount of each sample was loaded into each well of 4–12% NuPage Bis–Tris gels (Invitrogen) and electrophoresis was performed using a dissociation running buffer MOPS (Thermo Fisher, Waltham, MA, USA), at 180 V for around 1 h. Next, proteins were transferred to a PVDF membrane by electrophoresis at 350 mAmp for 1 h 20 min at 4 °C, subsequently blocked with 5% milk or BSA in PBS supplemented with 0.1% Tween-20 (PBST) for 1 h at room temperature (RT) and thereafter incubated with primary antibodies overnight at 4 °C. The primary antibodies used in this study were mouse monoclonal ATRX (Santa Cruz, Dallas, TX, USA, sc-55584), mouse monoclonal p53 (Santa Cruz, sc-126), rabbit monoclonal p21 (Cell Signaling, Danvers, MA, USA, 2947), mouse vinculin (Sigma, V9131) and mouse monoclonal β-actin (Sigma, A5441). On the following day, membranes were washed in PBST, incubated with the appropriate HRP-conjugated secondary antibodies for 1 h at RT, then washed again and stained with the chemiluminescent substrate AmerSham™ ECL™ Western Blotting Detection Reagents (GE Healthcare, Chicago, IL, USA). Images were acquired on an AmerSham™ Imager 600 (GE Healthcare Life Sciences). Goat anti-rabbit immunoglobulin G (IgG) (Sigma, A6667) and goat anti-mouse IgG (Sigma, A4416) were used as secondary antibodies.

### 2.4. High-Throughput Microscopy

Images were acquired using the ScanR acquisition software 3.2.0 (r4066) ×64 (Olympus, Johann-Krane-Weg, Munich, Germany) controlling a motorized Olympus IX-81 wide-field microscope. Olympus UPLSAPO 10x/0.4 NA objective was used. Single plane images corresponding to Z positions of maximal DAPI signal were acquired. At least nine images were acquired per well. Each fluorophore emission was collected separately and both images were acquired exactly in the same space conditions. The number of cells and mean signal intensity were analyzed and quantified with the ScanR analysis software (Olympus).

### 2.5. Analysis of the Screen Data

ATRX WT and ATRX KO HeLa clones were mixed. The mixed population was seeded on μCLEAR 96-well plates (Greiner Bio-One, Frickenhausen, Germany) at a density of 4000 cells/well. After 24 h, cells were treated with the compounds of the FDA-approved APExBIO drug library (L1021), with a final concentration of 10 µM and for a period of 48 h by adding one compound per well. DMSO was used as control. The number of cells from each condition was analyzed with ScanR analysis software 3.2.0 (r4066) ×64 (Olympus). Briefly, cell populations were selected according to their emitted fluorescence corresponding to ATRX staining, as WT (fluorescent, ATRX positive) and KO (non-fluorescent, ATRX negative) from each condition for the primary screen, and according to their tdTomato (ATRX positive) or GFP signal (ATRX negative) for the secondary screen. DAPI was used to identify the total number of cells. Drug-treated wells with <190 WT alive cells were excluded from further analysis. The cell viability was assessed with the following formula:

$$\mathrm{Cellular}\;\mathrm{viability}=\frac{n{}^{\circ}\;\mathrm{of}\;\mathrm{ATRX}\;\mathrm{WT}\;\mathrm{cells}\;\left(\mathrm{drug}\right)/nº\;\mathrm{of}\;\mathrm{ATRX}\;\mathrm{WT}\;\mathrm{cells}\;\left(\mathrm{DMSO}\right)}{nº\;\mathrm{of}\;\mathrm{ATRX}\;\mathrm{KO}\;\mathrm{cells}\;\left(\mathrm{drug}\right)/nº\;\mathrm{of}\;\mathrm{ATRX}\;\mathrm{KO}\;\mathrm{cells}\;\left(\mathrm{DMSO}\right)}$$

Drugs causing the same effect in the WT and KO clones scored a ratio of 1; drugs with a higher toxicity for the KO clones compared to the WT clones scored >1; and drugs with a higher toxicity for the WT clones compared to the KO clones scored <1.

### 2.6. Immunofluorescence

For all immunostaining, cells were seeded on μCLEAR 96-well plates. Then, cells that were treated as required were fixed with 4% formaldehyde (VWR Chemicals, Radnor, PA, USA) for 15 min at RT and permeabilized with 0.5% Triton X-100 (*v/v*) in PBS for 10 min. Cells were washed twice with PBST and blocked with 3% BSA (Sigma) in PBST for 30 min and then labeled with primary antibody at 4 °C overnight. The primary antibodies used in this study were ATRX (Santa Cruz, sc-55584) and γH2AX (MilliporeSigma, St. Louis, MO, USA, 05-636). Next, wells were washed in PBST and fluorescence-tagged secondary antibody (Alexa Fluor™ Goat Anti-mouse IgG 488 (Invitrogen)) that was added for 2 h at RT in the dark. DAPI was used for nuclear staining. The DNA replication rate was determined by EdU incorporation using Click-iT technology following the manufacturer’s instructions (Life Technologies). Briefly, EdU was added 30 min prior to fixing the cells and click chemistry was performed prior to blocking. A dilution of 1 mM ascorbic acid was prepared fresh, and the click-it reaction mix was performed by mixing PBS, CuSO_4_, Azide 647 and ascorbic acid in the given order, which was then added to each well for 1 h at RT in the dark.

### 2.7. Lentivirus Synthesis

HEK-293 T cells were used for the synthesis of third generation lentiviruses containing a tdTomato vector or a GFP vector. Then, 6 × 10^6^ of cells were reverse transfected using lipofectamine 2000 with 10 μg of the plasmid of interest and the plasmids coding for the lentivirus packaging components (6.5 µg pMMDLRRE, 2.5 µg PRSVREV and 3.5 µg PMDGVSVG) (#12251, #12253 and #12259, respectively, addgene). Forty-eight hours post-transfection, the viruses were filtered (0.25 µm) and collected.

### 2.8. Cell Infection

Cells were plated in 6-well dishes a day prior to infection. The cells were infected with different amounts of lentivirus containing the vector of interest and 10 µg/mL of polybrene to facilitate the infection. HeLa ATRX WT clones 1 and 2 were infected with a medium and high titer of lentivirus containing tdTomato plasmid, respectively; and HeLa ATRX KO clones 3, 4 and 5 were infected with a low, medium or high titer of lentivirus containing GFP plasmid, respectively. Furthermore, the U-251 (EV and ATRX KO) cells were infected with lentivirus containing short hairpin RNA (shRNA) targeting *TP53* (kindly provided by Prof. Oscar Fernandez-Capetillo) or the shRNA control of pLKO.1 (#8453, addgene). The following day, the infection efficiency was assessed by a fluorescent microscope (Olympus).

### 2.9. Drug Inhibitors

Ibrutinib (A3001), niclosamide (B2283), pazopanib (A3022), nintedanib (A8252), sunitinib (B1045), sorafenib (A3009), temozolomide (B1399) and CP-673451 (B2173) were purchased from APExBIO. 

### 2.10. Cell Viability Analysis

All the cell lines used for the cell viability analysis were seeded with a density of 4000 cells/well in μCLEAR 96-well plates. The following day, drugs were added in technical triplicates with the desired concentration and time. Subsequently, cells were fixed with 4% formaldehyde (VWR Chemicals) for 15 min at RT and stained for DAPI. Images were acquired with ScanR acquisition software with a 10×/0.4 NA objective and quantification of the number of cells was performed with ScanR analysis software. DMSO-treated cells were used as control. Cell viability was assessed by dividing the number of drug-treated cells by the number of DMSO-treated cells.

### 2.11. Dose–Response Curves

U-251 (EV and ATRX KO) high-grade glioma cells (4000 cells/well) or patient-derived GBM cells (8000 cells/well) were seeded in μCLEAR 96-well plates. On the following day, the medium was replaced with fresh medium containing increasing doses of the desired drug. After 48 h cells were fixed with 4% formaldehyde (VWR Chemicals) for 15 min at RT and stained for DAPI. Images were acquired with ScanR acquisition software with a 10×/0.4 NA objective and quantification of number of cells was performed with ScanR analysis software. The log of each known concentration in the dilution series (x-axis) was plotted against the number of cells (expressed as percentage) for that concentration (y-axis). The resulting dose–response curves were fit using GraphPad Prism. Drug’s potency (IC50 value) was determined by non-linear regression analysis of the resulting dose–response curve. Each dose–response curve was performed in biological triplicates (with technical triplicates for each experiment) and a representative replicate is shown in the manuscript.

### 2.12. Colony Formation Assay

U-251 (EV and ATRX KO) high-grade glioma cells were seeded in 10 cm plates at a density of 2000 and 3500 cells, respectively. Four days after seeding, the medium was replaced with fresh-medium containing sunitinib at 1.25 μM, 2.5 μM, 5 μM or vehicle. After 6 days of treatment, when colonies were visible, cells were washed with PBS, fixed and stained with a solution containing 20% absolute ethanol and 0.5% crystal violet in water for 1 h at RT. Cells were washed twice with water and after allowing to air dry, images were acquired and the number of colonies was quantified using the Analyze Particles plugin of ImageJ software.

### 2.13. Statistical Analysis

Statistical analyses were performed using GraphPad Prism 8. The significance was determined by either unpaired *t*-test or Fisher’s exact test. The *p*-values are indicated in each graph and in the figure legends.

## 3. Results 

### 3.1. Identification of FDA-Approved Compounds That Selectively Kill ATRX-Deficient Cells

**Figure 1:**
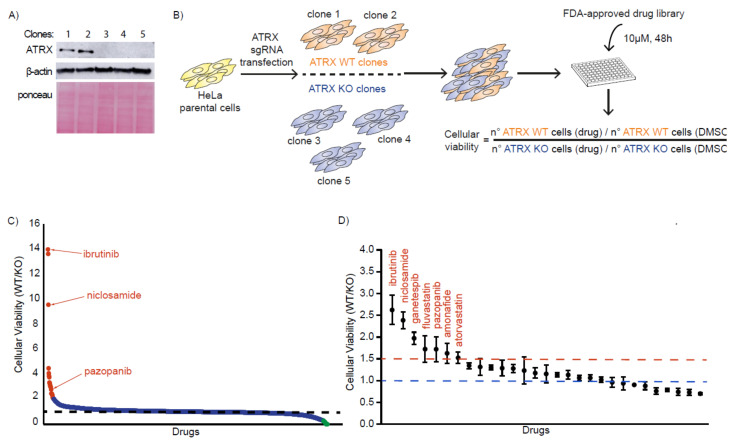
FDA-Approved drug screen identifies compounds synthetically lethal with ATRX-deficiency. (**A**) Immunoblotting of HeLa clones generated by CRISPR. (**B**) Drug screen flowchart. (**C**) Primary drug screen. Cell viability (WT/KO) after 48 h of drug treatment. Each point represents one drug. Red dots indicate drugs with a 2-fold higher lethality effect in the ATRX KO clones compared to WT clones. Green dots indicate compounds that induce a 2.5-fold higher toxicity in ATRX WT clones compared to ATRX KO clones. (**D**) Secondary drug screen. Cell viability (WT/KO) after 48 h of drug treatment of the 29 top hits derived from the primary screen. Each point represents one drug. Data shown correspond to technical triplicates. Means and SDs are indicated.

To create isogenic cell lines that either possess or lack ATRX, we generated HeLa ATRX knockout (KO) cells using CRISPR/Cas9. We designed a gRNA targeting the fourth coding exon of the *ATRX* gene and obtained three independent ATRX KO clones. Two HeLa ATRX WT clones were obtained in parallel and used as a control for further experiments (Figure 1A and Figure S1A). DNA replication was assessed by EdU (5-Ethynyl-2′-deoxyuridine) incorporation. All the clones proliferated at similar rates, although the number of replicating cells slightly decreased in two ATRX KO clones as shown by the percentage of EdU-positive cells (Figure S1B). We also assessed the levels of H2AX phosphorylation (γH2AX) as a marker of DNA damage. The three ATRX KO clones exhibited higher levels of γH2AX as compared to the WT clones indicating that the loss of ATRX leads to increased DSBs formation and genome instability, as reported previously (Figure S1C) [8,25]. Recent studies have revealed that the use of CRISPR to generate KO cell lines often results in mutations in *TP53* [26,27]. Therefore, we assessed the p53 status in all of the HeLa clones. We observed that all of the ATRX WT and ATRX KO cell lines used in this study were p53 proficient and were able to activate p21 upon DNA damage (Figure S1D). 

To screen for drugs that are synthetic lethal with ATRX loss, we used the FDA-approved APExBIO drug library, which contains 1496 compounds. We performed the screen using a mixed population of cells (mixed in 1 to 1 ratio) containing the two ATRX WT and three ATRX KO clones that could be discriminated by ATRX immunofluorescence (IF) (Figure S1E). Cells were treated with the compounds at 10 µM for 48 h and cell viability was assessed by DAPI staining combined with high-content microscopy (Figure 1B). We evaluated the effect of the drugs on the viability of WT and ATRX KO clones by calculating the ratio of the number of WT cells by the number of KO cells compared to their DMSO control cells (Figure 1B,C). From this screen, we identified 29 compounds (red-colored drugs in Figure 1C and Table S1) that caused at least a 2-fold higher toxicity to the KO cells compared to the WT cells.

In addition, we also identified 37 compounds that induced at least a 2.5-fold higher lethality to the ATRX WT cells compared to the ATRX KO cells (green-colored drugs in Figure 1C and Table S1). However, we chose to focus on the drugs that trigger death in ATRX-deficient cells in this study, and further analyses should be performed to validate and characterize the compounds that appear to be less toxic for ATRX KO cells.

### 3.2. ATRX-Deficient Cells Show Increased Sensitivity to BTK, STAT3, and RTK Inhibitors

To validate the effect of the 29 compounds identified in the initial screen, we performed a secondary screen whereby the two ATRX WT and three ATRX KO HeLa clones were labeled with different fluorescent protein markers. The two WT clones (clones 1 and 2) were infected with different titers of lentivirus expressing fluorescent tdTomato protein and the three ATRX KO clones (clones 3, 4 and 5) with different titers of lentivirus expressing GFP (Figure S1F). This strategy allowed us to distinguish the individual tdTomato-ATRX WT and GFP-ATRX KO clones within the mixed population (Figure S1G). Importantly, replication among the clones was not affected after the lentivirus infection and the GFP-ATRX KO clones showed increased levels of γH2AX as compared to their tdTomato-ATRX WT counterparts, as seen for the unlabeled clones used for the initial screen (Figure S1H,I). We employed a similar strategy for the secondary screen using a mixed population containing the five labelled clones; cells were treated with 10 µM of the 29 selected compounds for 48 h. Assessment of cell viability using high-content microscopy revealed that seven out of the twenty-nine analyzed compounds showed a toxicity of at least 1.5-fold higher in the ATRX KO clones as compared to the ATRX WT clones (Figure 1D). Among these seven validated hits, we further characterized the inhibitors targeting BTK (ibrutinib), STAT3 (niclosamide), and RTK (pazopanib), as previous studies have indicated the potential of these drugs to treat high-grade glioma [28,29,30,31].

**Figure 2:**
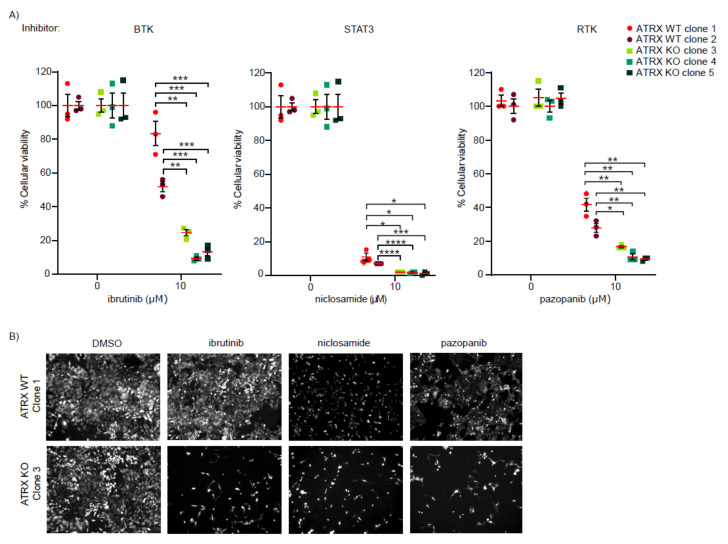
ATRX-deficient HeLa cells are susceptible to BTK, STAT3 and RTK inhibitors. (**A**) Cell viability of tdTomato-ATRX WT and GFP-ATRX KO HeLa clones after 72 h of treatment with the indicated drugs and concentrations compared to DMSO controls. Data shown correspond to technical triplicates. Means and SEMs are shown. Statistics for significant conditions are shown. Significance was assessed by unpaired *t*-test. * *p* ≤ 0.05; ** *p* ≤ 0.01; *** *p* ≤ 0.001; **** *p* ≤ 0.0001. (**B**) Representative images of clone 1 (ATRX WT) and clone 3 (ATRX KO) after 72 h of treatment with either DMSO or the indicated drugs at a concentration of 10 μM.

To further validate the effect of ibrutinib, niclosamide, and pazopanib, we treated the tdTomato ATRX WT and GFP-ATRX KO HeLa clones individually at 10 µM and assessed cell viability at different time points (24 h, 48 h, and 72 h). The three ATRX-deficient HeLa clones were more sensitive to ibrutinib, niclosamide, and pazopanib (Figure 2A,B and Figure S2A). The higher toxicity of the drugs for ATRX-deficient clones was observed at 48 h and 72 h of drug treatment at 10 µM (Figure 2A and Figure S2A). Finally, we tested three additional FDA-approved RTKi (nintedanib, sunitinib, and sorafenib), all of which target PDGFR. Nintedanib and sunitinib caused higher cell toxicity in all the ATRX KO HeLa clones compared to the HeLa ATRX WT counterparts when used at 6 µM for 48 h (Figure S2B). In addition, treatment with sorafenib in the same conditions induced higher toxicity in two out of the three ATRX KO HeLa clones compared to the HeLa WT cells, although not all the conditions were statistically significant. Taken together, our data indicate that BTKi, STAT3i, and multitarget RTKi are toxic to ATRX-deficient cells.

### 3.3. ATRX KO High-Grade Glioma Cells Are Sensitive to RTK and PDGFR Inhibitors

Given the relevance and frequency of ATRX mutations in high-grade glioma patients, we sought to validate the effect of BTK, STAT3, and RTK inhibitors in glioma cell lines derived from adult malignant glioma (MOG-G-UVW and U-251) and pediatric malignant glioma (SF188) patients. We used CRISPR/Cas9 engineered ATRX KO high-grade glioma cell lines and empty vector (EV) control cell lines (Figure S3A,B) that were generated in a previous study [10]. The ATRX KO malignant glioma cell lines incorporated EdU similar to the ATRX WT cell lines, indicating that ATRX KO cells replicate and proliferate at normal rates (Figure S3C), which is important to assess the toxicity caused by the drug treatments. However, unlike the HeLa ATRX KO clones, the ATRX KO glioma cell lines used in this study showed similar levels of γH2AX as compared to the EV cells (Figure S3D). 

**Figure 3:**
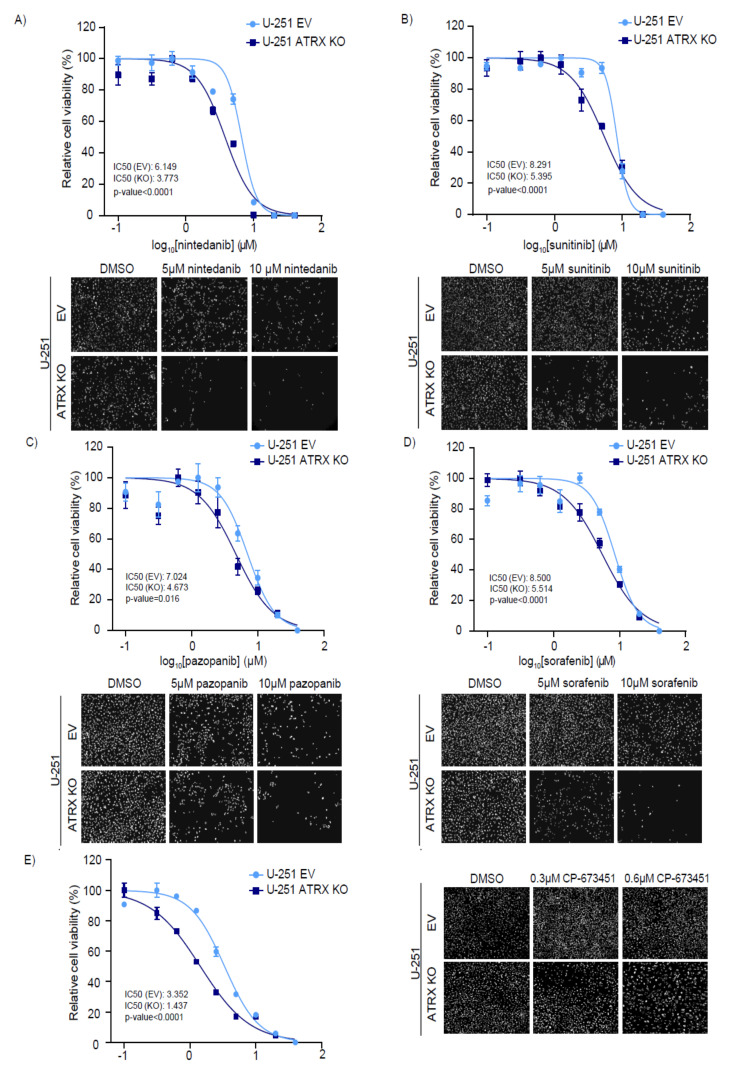
ATRX-deficient high-grade glioma cells are sensitive to multi-targeted RTK and specific PDGFR inhibitors. (**A**–**E**) Relative cell viability of U-251 (EV and ATRX KO) high-grade glioma cell lines treated with increasing doses of the indicated drug. The IC50 values were calculated from the dose–response curve determined by GraphPad Prism. Data shown correspond to a representative experiment (with technical triplicates) out of three biological replicates. SEMs from each data point are indicated. Significance was assessed by F-test and the *p*-value for each dataset is shown. Representative images of EV and ATRX KO cells after 48 h of treatment with DMSO or the indicated drug concentrations are shown.

We treated MOG-G-UVW, U-251, and SF-188 (EV and ATRX KO) high-grade glioma cell lines with different concentrations (5 µM and 10 µM) of BTK, STAT3, and RTK inhibitors for 48 h in order to assess cell viability. Ibrutinib (BTKi) and niclosamide (STAT3i) did not show a consistent increased toxicity in the ATRX-deficient glioma cells as compared to their ATRX-proficient counterparts (Figure S3E,F), suggesting that the sensitivity of ATRX-deficient cells to these inhibitors may be influenced by other factors. Interestingly, the four RTKi tested induced higher toxicity to a different extent in the three ATRX KO high-grade glioma cell lines when used at 5 µM and 10 µM (Figure S4A–D). To further confirm this finding, we performed additional experiments with the U-251 cell lines. U-251 cells (EV and ATRX KO) were treated with increasing concentrations of the four RTKi to determine their IC50 values. In line with the previous results, the U-251 ATRX KO cells showed increased sensitivity to the four RTKi tested with an IC50 at least 1.5-fold lower than their ATRX WT counterpart cells (Figure 3A–D).

**Figure 4:**
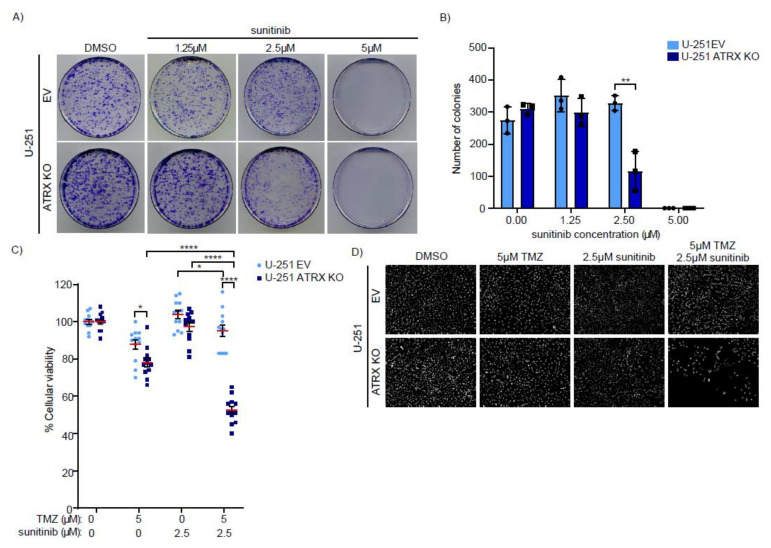
ATRX-deficient high-grade glioma cells are sensitive to combinatorial treatments of TMZ and sunitinib. (**A**,**B**) Clonogenic assays of U-251 (EV and ATRX KO) high-grade glioma cells exposed to the indicated doses of sunitinib. Colonies were quantified using Analyze Particles plugin of ImageJ software. Data shown correspond to biological triplicates. Means and SDs are indicated. Statistics for significant conditions are shown. Significance was assessed by unpaired *t*-test. ** *p* ≤ 0.01. (**C**) Cell viability of U-251 (EV and ATRX KO) high-grade glioma cells after 48 h of treatment with TMZ and/or sunitinib at the indicated concentrations compared to DMSO controls. Data shown correspond to four independent experiments. Mean and SEMs are indicated. Statistics for significant conditions are shown. Significance was assessed by unpaired *t*-test. * *p* ≤ 0.05; **** *p* ≤ 0.0001. (**D**) Representative images of U-251 (EV and ATRX KO) high-grade glioma cells after 48 h of treatment with either DMSO or the indicated drugs and concentrations.

Furthermore, we tested the effect of RTKi by performing clonogenic assays. Sunitinib treatment significantly reduced clonogenic survival of U-251 ATRX KO cells compared to WT cells (Figure 4A,B),further validating the toxicity of RTKi for ATRX-deficient high-grade glioma cells.

The four RTKi used (nintedanib, sunitinib, pazopanib, and sorafenib) are very potent inhibitors of PDGFR but also inhibit other RTK, such as VEGFR or FGFR to a variable extent [32]. In order to decipher whether ATRX-deficient cells are sensitive to PDGFRi, we assessed the sensitivity of MOG-G-UVW, U-251, and SF-188 (EV and ATRX KO) high-grade glioma cell lines to the potent and selective PDGFRi, CP-673451. The three isogenic (EV and ATRX KO) glioma cell lines were treated with different concentrations (0.3 µM and 0.6 µM) of CP-673451 for 48 h to assess cell viability. We found that all the ATRX KO high-grade glioma cell lines are more sensitive to CP-673451 at 0.6 µM compared to their WT counterparts (Figure S4E). In addition, U-251 (EV and ATRX KO) were treated with increasing concentrations of CP-673451 to determine their IC50 values. Importantly, U-251 ATRX KO cells have an IC50 of 2-fold lower when treated with CP-673451 than the U-251 EV cells (Figure 3E), indicating that specific inhibition of PDGFR leads to higher cell toxicity in ATRX-deficient cells.

### 3.4. ATRX KO High-Grade Glioma Cells Are Sensitive to Combinatorial Treatments of TMZ and PDGFRi

The current standard therapy for GBM includes TMZ and radiotherapy. In addition, it has been previously shown that ATRX KO cells are more sensitive to TMZ [33]. Therefore, we explored the effect of combining TMZ and PDGFRi on ATRX-deficient high-grade glioma cells. To this end, we assessed the toxicity of a combined treatment consisting of low doses of TMZ and sunitinib or CP-673451 in U-251 EV and ATRX KO cells.

Treatments with low doses of either TMZ, sunitinib or CP-673451 resulted in a slightly decreased cell viability in the ATRX KO cells as compared to WT cells (Figure 4C,D and Figure S5A,B). However, the combinatorial treatments with TMZ and sunitinib or CP-673451 led to a more pronounced decreased viability in the ATRX KO cells, (Figure 4C,D and Figure S5A,B).

These data suggest that the use of combinatorial treatments with TMZ and RTKi or PDGFRi may increase the therapeutic window of opportunity in GBM patients with *ATRX* mutations.

### 3.5. Patient-Derived GBM Cells with ATRX Mutations Are Sensitive to RTK and PDGFR Inhibitors

**Figure 5:**
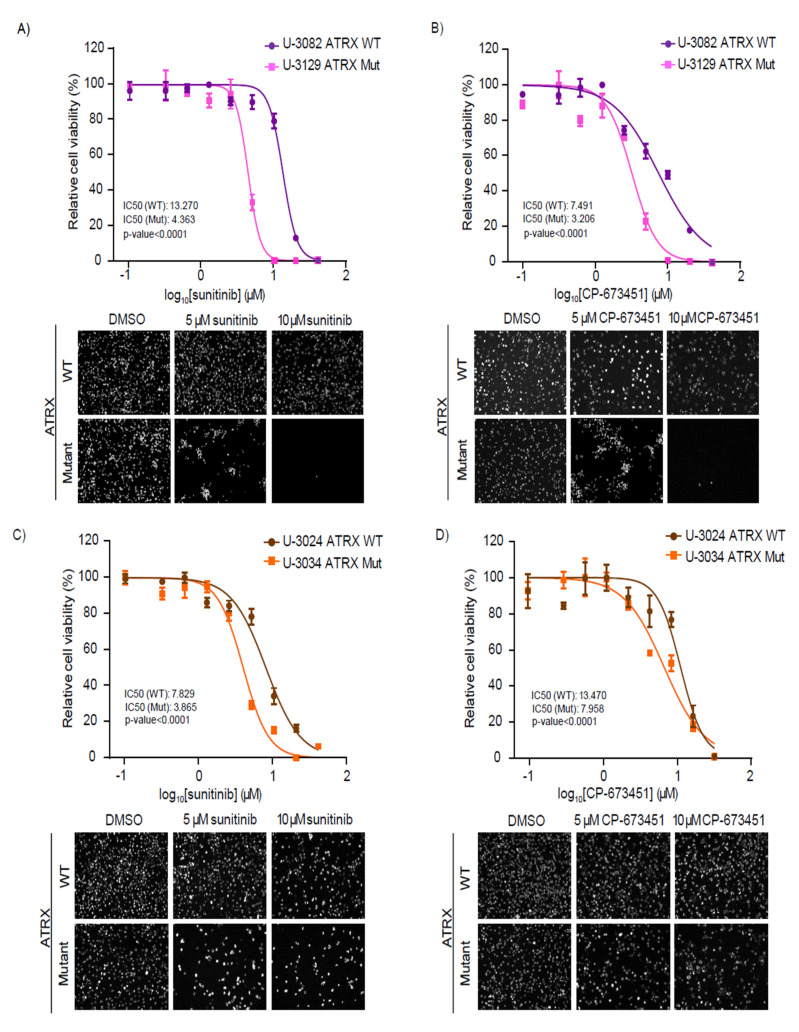
*ATRX*-mutant patient-derived high-grade glioma cells are sensitive to RTK and PDGFR inhibitors. (**A**–**D**) Relative cell viability of ATRX WT (U-3082 and U-3024) and ATRX Mutant (U-3129 and U-3034) high-grade glioma cell lines treated with increasing doses of the indicated drug. The IC50 values were calculated from the dose–response curve determined by GraphPad Prism. Data shown correspond to a representative experiment (with technical triplicates) out of three biological replicates. SEMs from each data point are indicated. Significance was assessed using F-test and the *p*-value for each dataset is shown. Representative images of ATRX WT and ATRX Mutant cells after 48h of treatment with DMSO or the indicated drug concentrations are shown.

Next, we aimed to validate these findings in cells derived from adult malignant glioma patients harboring somatic *ATRX* mutations. We used patient-derived GBM cells from the human glioblastoma cell culture (HGCC) biobank (https://www.hgcc.se/, accessed on 21 March 2022). These cells were obtained from surgical samples of human adult GBM patients and cultured as described in Xie et al., 2015 [34]. We compared the effect of sunitinib and the more specific PDGFRi CP-673451 in two cell lines harboring mutations in *ATRX* (U3129 and U3034) and two without them (U3082 and U3024). The U3129 and U3034 patient-derived glioma cells used in this study harbor intronic and missense mutations in *ATRX* (https://www.hgcc.se/, accessed on 21 March 2022), which, besides potential alterations to protein functionality, lead to reduced ATRX levels (Figure S5C,D). Cells were grown and studied in pairs grouped according to their molecular subtype, proneural GBM (U-3082 and U-3129), and mesenchymal GBM (U-3024 and U-3034). These cells were treated with increasing concentrations of sunitinib or CP-67345 and IC50 s were calculated as described previously. Cells harboring *ATRX* mutations were more sensitive to sunitinib and CP-673451 than *ATRX* WT cells, showing an IC50 of at least 1.6-fold lower (Figure 5A–D). 

*ATRX* mutations usually co-occur with mutations in the *TP53* gene in high-grade glioma tumors, which could influence the response to these drugs [35,36,37]. The U-251 cell line used in this study harbors mutations in the *TP53* gene [10]. However, it should be noted that we detected a functional p53 response upon DNA damage in terms of p53 accumulation and p21 activation in the three isogenic cell lines (Figure S6A). We tested whether p53 depletion could influence the sensitivity of U-251 (EV and ATRX KO) cells to the RTKi sunitinib and nintedanib. Of note, cells infected with the control shRNAs exhibited high p21 and p53 basal levels, probably due to the stress caused by the recent viral infection. Nevertheless, p53-depleted U-251 ATRX KO cells, generated by p53 shRNA transduction, were also more sensitive to RTKi than p53-depleted ATRX WT cells (Figure S6B,C).

## 4. Discussion

Given the high prevalence of *ATRX* mutations in cancer, particularly in gliomas (reviewed in [15]), identifying drugs highly toxic for ATRX-deficient cells may lead to clinical applications. Thus, we performed high-content microscopy-based drug screens using ATRX WT and ATRX KO isogenic HeLa cell lines generated by CRISPR/Cas9. Of note, we used an FDA-approved drug library, hence the biosafety of the compounds was already tested and the findings from this screen could be rapidly implemented in the clinic. We used two pipelines to perform drug screens, in both cases pooling ATRX WT and ATRX KO cells in the same wells, which facilitated the analysis of a high number of drugs and the direct comparison of the effect of the drugs. In the primary screen, the p96-well plates were stained with an ATRX antibody to discriminate between ATRX WT and KO cells after 48 h of drug treatment. The top hits validated in a secondary screen where the isogenic clones were GFP/tdTomato-labeled by lentiviral transduction prior to the screen. This second strategy allows to quantify the effect of the drugs on individual clones at different time points and doses in one single experiment. 

We focused on the validation of the drugs that caused higher toxicity to ATRX KO cells compared to WT cells. However, the drugs that caused lower toxicity could also provide interesting clinical information. For instance, patients with *ATRX* mutations could be refractory to treatments with those drugs. Since ATRX is a regulator of therapy-induced senescence [11], some of the drugs causing less toxicity in ATRX KO cells may act by inducing senescence in ATRX WT cells, and therefore, in that scenario, ATRX-deficient cells might present a proliferative advantage compared to the ATRX-proficient cells. 

Among the 29 top hits identified in the primary screen, seven compounds were validated in the secondary screen, also using HeLa cells. Next, we studied three of these drugs (ibrutinib, niclosamide, and pazopanib) in isogenic ATRX KO high-grade glioma cell lines previously generated by CRISPR/Cas9 [10]. Ibrutinib and niclosamide have shown positive results in the treatment of glioma cells, regardless of the ATRX status [28,29,31]. Furthermore, ibrutinib and pazopanib have been used in clinical trials for GBM patients (NCT03535350, NCT00459381). However, only the RTKi pazopanib led to increased toxicity in ATRX KO high-grade glioma cells compared to the WT counterparts. This suggests that the ATRX-related sensitivity to STATi and BTKi is influenced by other factors such as the cellular genetic background and the cell type from which the tumor originates. It would be worth analyzing the sensitivity to these drugs in other cancer types presenting *ATRX* mutations, such as hepatocellular carcinoma, pancreatic neuroendocrine tumors, and neuroblastomas, and in cells with different genetic backgrounds. 

Three different ATRX KO high-grade glioma cell lines showed sensitivity to pazopanib and three additional RTKi (sorafenib, nintedanib, and sunitinib), all of which target PDGFR and other RTKs. Interestingly, a specific PDGFRi (CP-673451) also produced higher toxicity in ATRX KO cells, indicating that PDGFR is the main target responsible for this ATRX-related sensitivity. Enhanced PDGFR signaling is associated with the progression of several pathologies, including tumorigenesis [38], hence many anticancer therapies are based on targeting PDGFR signaling. The overexpression of the PDGFR alpha subunit has been reported to correlate with *ATRX* mutations in cancer cells [16,17]. This may suggest a direct or indirect compensatory relationship between these two events, which may explain the higher toxicity of PDGFRi in ATRX-deficient high-grade glioma cells. Furthermore, the increased sensitivity of ATRX-deficient cells to RTKi (i.e., sunitinib) has been recently shown in other cancer cell types, such as neuroblastoma, reinforcing our findings [39]. In addition, our study shows that combinatorial treatments of RTKi or PDGFRi and TMZ (the current standard of care treatment for GBM patients) might be beneficial for patients with tumors harboring *ATRX* mutations, which is in line with previous studies showing that TMZ inhibits glioma formation and increases the chemosensitivty of ATRX-deficient gliomas *in vivo* [33]. Importantly, there are several clinical trials using similar combinatorial treatments for GBM (i.e., NCT00597493, NCT00544817), and it has been shown that combinatorial treatments of RTKi plus TMZ can be safely administered to these patients [40,41].

We found that patient-derived GBM cells harboring somatic ATRX mutations are also more sensitive to RTK and PDGFR inhibitors, supporting that high-grade glioma patients harboring *ATRX* mutations could respond better to these drugs. Most ATRX mutations found in glioma patients are truncating mutations leading to the complete loss of the functional ATRX protein. Thus, the isogenic ATRX KO cells used in this study resemble the ATRX status in most cancer patients with *ATRX* mutations. However, some patients present *ATRX* missense mutations, which are predicted as pathogenic but are less characterized. Indeed, the mutations identified in the patient-derived glioma cells used in this study (U3129 and U3034) are intronic and missense mutations that lead to reduced ATRX levels. All ATRX KO high-grade glioma cell lines used in this study were more sensitive to RTKi and PDGFRi than ATRX WT cells, independently of the type of mutation in *ATRX*. Patients with ATRX-deficient high grade glioma tumors often harbor mutations in other genes, such as *TP53* or *IDH1*, which could influence the response to these drugs [35,36,37]. The high-grade glioma cell lines used in our study do not harbor mutations in the *IDH1/2* genes [10,34,42]. Since secondary GBM frequently harbor mutations in both, *IDH1* and *ATRX* genes [43], future studies should address the toxicity of RTKi and selective PDGFRi (with and without TMZ) in cells with this relevant mutational background. On the other hand, all the patient-derived GBM cells and two of the isogenic high-grade glioma cell lines used in our study, U-251 and SF188, harbor mutations in the *TP53* gene [10,34], and all the ATRX-deficient cell lines tested were sensitive to RTKi and PDGFRi irrespective of their *TP53* status. Therefore, these inhibitors could also be effective in treating GBM patients harboring *TP53* and *ATRX* co-occurrent mutations. 

## 5. Conclusions

In this study, we have demonstrated that ATRX deficient cells, including patient-derived high-grade glioma cell lines, are particularly sensitive to RTKi (pazopanib, nintedanib, sunitinib, sorafenib) and to a specific PDGFRi (CP-673451). Besides, this sensitivity was enhanced when RTKi or PDGFRi were combined with TMZ (current standard of care in glioblastoma treatment). Importantly, there are several clinical trials using similar combinatorial treatments for glioblastoma (i.e., NCT02928575, NCT00597493, NCT00544817, NCT02331498). Therefore, based on our findings and the frequent presence of *ATRX* mutations in patients who suffer secondary GBM or anaplastic astrocytomas, we suggest that the analyses of these clinical trials data should consider the ATRX status.

## Data Availability

The data presented in this study are available on request to the corresponding author.

## Supplementary Materials

The following supporting information can be downloaded at: https://www.mdpi.com/article/10.3390/cancers14071790/s1, Figure S1: Characterization of HeLa ATRX-KO clones, Figure S2: Cellular viability of tdTomato ATRX WT and GFP ATRX KO HeLa clones upon treatment with BTK, STAT3 and RTK inhibitors Figure S3: CRISPR/Cas9 engineered MOG-G-UVW, U-251 and SF188 (EV and ATRX KO) high-grade glioma cells: ATRX expression, effect on EdU incorporation and γH2AX levels, and sensitivity to BTK and STAT3 inhibitors, Figure S4: Cell viability of MOG-G-UVW, U-251 and SF-188 (EV and ATRX KO) upon treatment with RTK and PDGFR inhibitors, Figure S5: ATRX-deficient high-grade glioma cells are sensitive to combinatorial treatments of TMZ and CP-673451, Figure S6: TP53 loss does not influence the toxicity of RTK inhibitors in ATRX-KO U-251 high-grade glioma cells Table S1. List of compounds identified in the first screen.

## Abbreviation

ALT	Alternative Lengthening of Telomeres
ATRX	alpha thalassemia mental retardation X-linked
BTK	Bruton’s tyrosine kinase
BTKi	inhibitors targeting BTK
CFS	common fragile sites
DAXX	death domain-associated protein 6
DMSO	dimethyl sulfoxide
DSB	double-strand break
EdU	5-Ethynyl-2′-deoxyuridine
EV	empty vector
FDA	food and drug administration
GBM	glioblastoma multiforme
GFP	green fluorescent protein
gRNA	guide RNA
H3.3	histone H3.3
HGCC	human glioblastoma cell culture
IDH1	isocitrate dehydrogenase 1
KO	knockout
PDGFR	platelet-derived growth factor receptor
PDGFRi	inhibitors targeting PDGFR
RTK	receptor tyrosine kinase
RTKi	inhibitors targeting RTK
shRNA	short hairpin RNA
STAT3	signal transducer and activator of transcription 3
STAT3i	inhibitors targeting STAT3
TMZ	temozolomide
TP53	tumor protein 53
WT	wild-type
γH2AX	histone H2AX phosphorylation
