# Supplementary material for: ATRX-Deficient High-Grade Glioma Cells Exhibit Increased Sensitivity to RTK and PDGFR Inhibitors
Source: Cancers (Basel). 2022 Mar 31;14(7):1790. doi: 10.3390/cancers14071790 (PMC8997088; doi:10.3390/cancers14071790)
Supplement: Supplementary file 1 [file cancers-14-01790-s001.zip › cancers-1619206-supplementary/cancers-1619206-supplementary.pdf]

cancers

Article

ATRX-Deficient High-Grade Glioma Cells Exhibit Increased Sensitivity to RTK and PDGFR Inhibitors

David Pladevall-Morera [1,†], María Castejón-Griñán [1,2,†], Paula Aguilera [1,2], Karina Gaardahl [1], Andreas Ingham [1], Jacqueline A. Brosnan-Cashman [3], Alan K. Meeker [3] and Andres J. Lopez-Contreras [1,2,*]

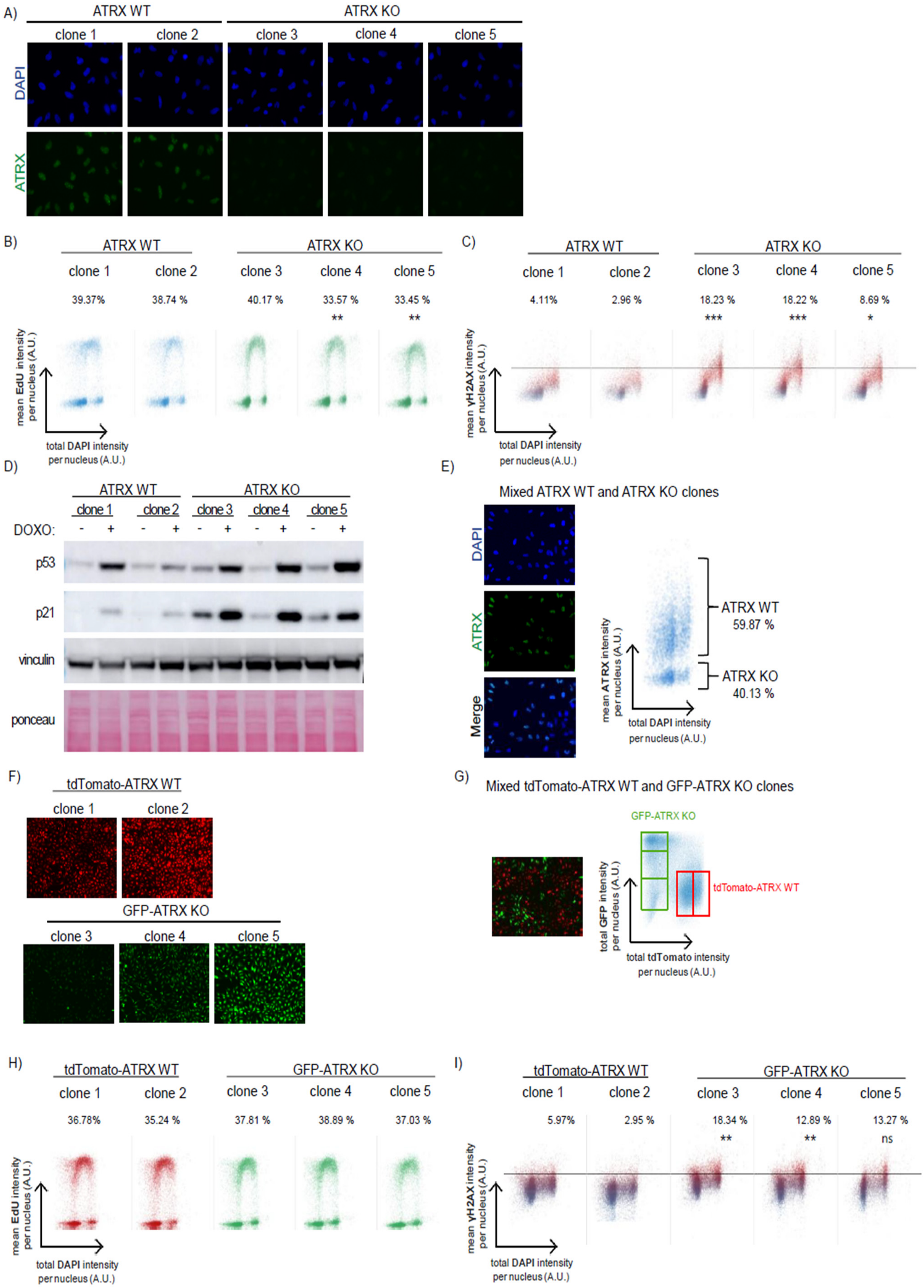
A)
ATRX WT
ATRX KO
clone 1
clone 2
clone 3
clone 4
clone 5
DAPI
ATRX
B)
ATRX WT
ATRX KO
clone 1
clone 2
clone 3
clone 4
clone 5
39.37%
38.74 %
40.17 %
33.57 %
33.45 %
**
**
mean EdU intensity per nucleus (A.U.)
total DAPI intensity per nucleus (A.U.)
C)
ATRX WT
ATRX KO
clone 1
clone 2
clone 3
clone 4
clone 5
4.11%
2.96 %
18.23 %
18.22 %
8.69 %
***
***
*
mean γH2AX intensity per nucleus (A.U.)
total DAPI intensity per nucleus (A.U.)
D)
ATRX WT
ATRX KO
clone 1
clone 2
clone 3
clone 4
clone 5
DOXO: - + - + - + - + - +
p53
p21
vinculin
ponceau
E)
Mixed ATRX WT and ATRX KO clones
DAPI
ATRX
Merge
mean ATRX intensity per nucleus (A.U.)
total DAPI intensity per nucleus (A.U.)
ATRX WT
59.87 %
ATRX KO
40.13 %
F)
tdTomato-ATRX WT
clone 1
clone 2
GFP-ATRX KO
clone 3
clone 4
clone 5
G)
Mixed tdTomato-ATRX WT and GFP-ATRX KO clones
GFP-ATRX KO
tdTomato-ATRX WT
total GFP intensity per nucleus (A.U.)
total tdTomato intensity per nucleus (A.U.)
H)
tdTomato-ATRX WT
GFP-ATRX KO
clone 1
clone 2
clone 3
clone 4
clone 5
36.78%
35.24 %
37.81 %
38.89 %
37.03 %
mean EdU intensity per nucleus (A.U.)
total DAPI intensity per nucleus (A.U.)
I)
tdTomato-ATRX WT
GFP-ATRX KO
clone 1
clone 2
clone 3
clone 4
clone 5
5.97%
2.95 %
18.34 %
12.89 %
13.27 %
**
**
ns
mean γH2AX intensity per nucleus (A.U.)
total DAPI intensity per nucleus (A.U.)

Figure S1. Characterization of HeLa ATRX-KO clones. (**A**) Assessment of ATRX KO clones by IF. (**B**) Quantitative image-based cytometry (QIBC) of HeLa clones was performed and total DAPI/mean EdU intensities were plotted in scatter diagrams showing replication profiles. Percentages of EdU positive cells and statistics for significant conditions are shown. Individual ATRX KO clones were compared to each ATRX WT clone. Significance was assessed by unpaired t test **$p \leq 0.01$. (**C**) QIBC of immunolabeled HeLa clones was performed and total DAPI/mean γH2AX intensities were plotted in scatter diagrams. Red dots indicate γH2AX positive cells. Percentage of γH2AX positive cells and statistics for significant conditions are shown. Individual ATRX KO clones were compared to each ATRX WT clone. Significance was assessed by unpaired t test *$p \leq 0.05$. ***$p \leq 0.001$ (**D**) Immunoblotting of the HeLa clones with the indicated antibodies. Cells were untreated or treated with doxorubicin (0.5μM) for 12h. (**E**) QIBC of immunolabeled HeLa clones was performed and total DAPI/mean ATRX intensities were plotted in scatter diagrams showing the two different populations consisting of ATRX WT and ATRX KO clones. Percentage of ATRX WT and ATRX KO cells is shown. (**F**) Representative images of the HeLa clones infected with different titers of lentivirus expressing fluorescent tdTomato protein (clones 1 and 2) or different titers of GFP (clones 3, 4 and 5). (**G**) QIBC of dTomato-ATRX WT clones and GFP-ATRX KO clones was performed and total tdTomato/total GFP intensities were plotted in scatter diagrams showing the different popula- tions. (**H**) QIBC of tdTomato-ATRX WT clones and GFP-ATRX KO clones was performed and total DAPI/mean EdU intensities were plotted in scatter diagrams showing replication profiles. Percentages of EdU positive cells and statistics for significant conditions are shown. Individual ATRX KO clones were compared to each ATRX WT clone. Significance was assessed by unpaired t test **$p \leq 0.01$. (**I**) QIBC of immunolabeled tdTomato-ATRX WT clones and GFP-ATRX KO clones was performed and total DAPI/mean γH2AX intensities were plotted in scatter diagrams. Red dots indicate γH2AX positive cells. Percentage of γH2AX positive cells and statistics for significant conditions are shown. Individual ATRX KO clones were compared to each ATRX WT clone. Significance was assessed by unpaired t test *$p \leq 0.05$;**$p \leq 0.01$;***$p \leq 0.001$.

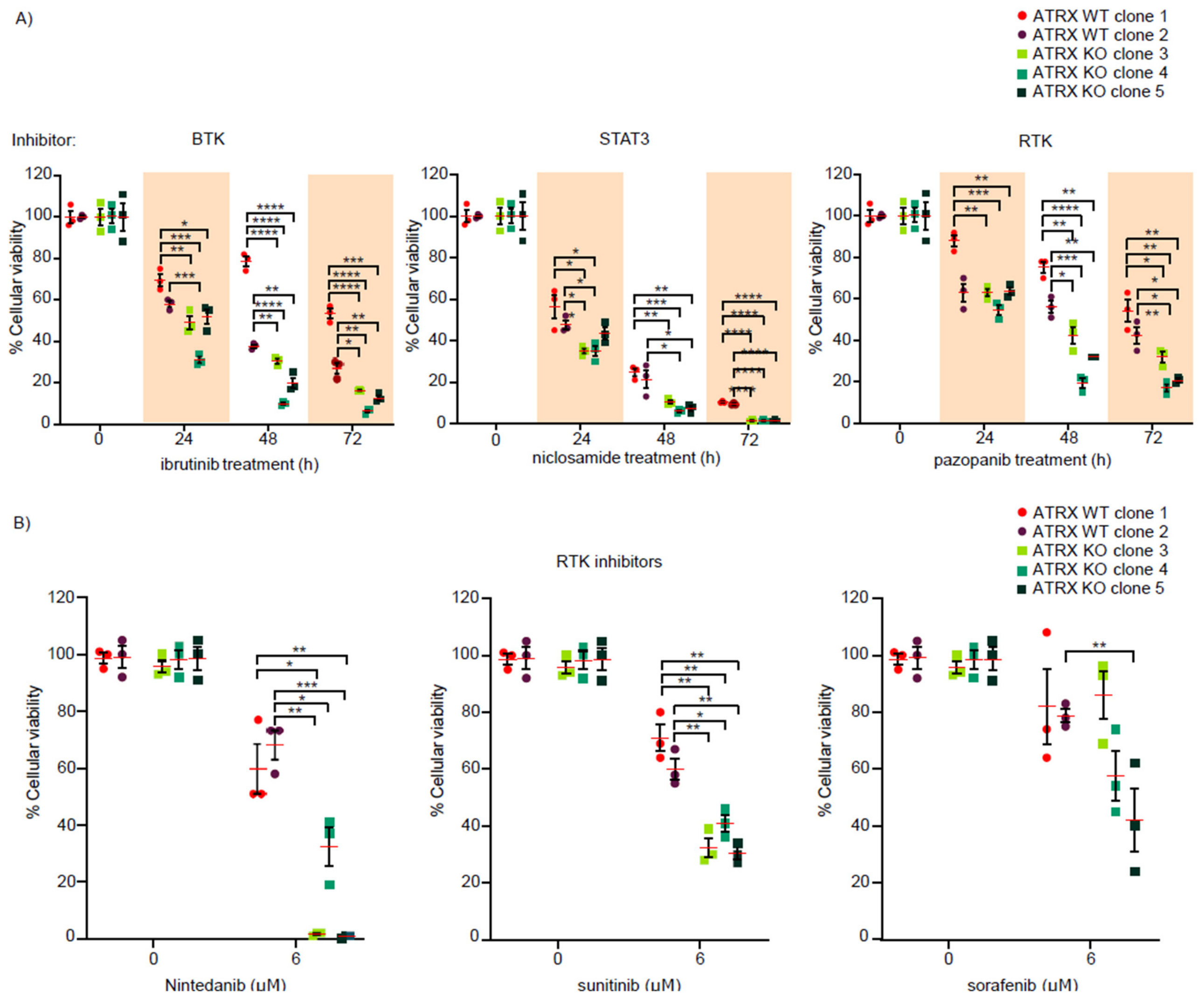

Figure S2. Cellular viability of tdTomato ATRX WT and GFP ATRX KO HeLa clones upon treatment with BTK, STAT3 and RTK inhibitors. (**A**) Cellular viability of tdTomato-ATRX WT and GFP-ATRX KO clones treated with 10μM of the indicat-ed drugs and for the indicated times compared to DMSO controls. Data shown corresponds to technical tripli- cates. Statistics for significant conditions, means and SEMs are shown. Significance was assessed by unpaired t-test. *p≤0.05; **p≤0.01; ***p≤0.001;****p≤0.0001. (**B**) Cellular viability of tdTomato-ATRX WT and GFP-ATRX KO clones after 48h of treatment with the indicated RTK inhibitors and concentrations compared to DMSO controls.Data shown corresponds to technical triplicates. Statistics for significant conditions, means and SEMs are shown. Signif- icance was assessed by unpaired t-test *p≤0.05; **p≤0.01; ***p≤0.001.

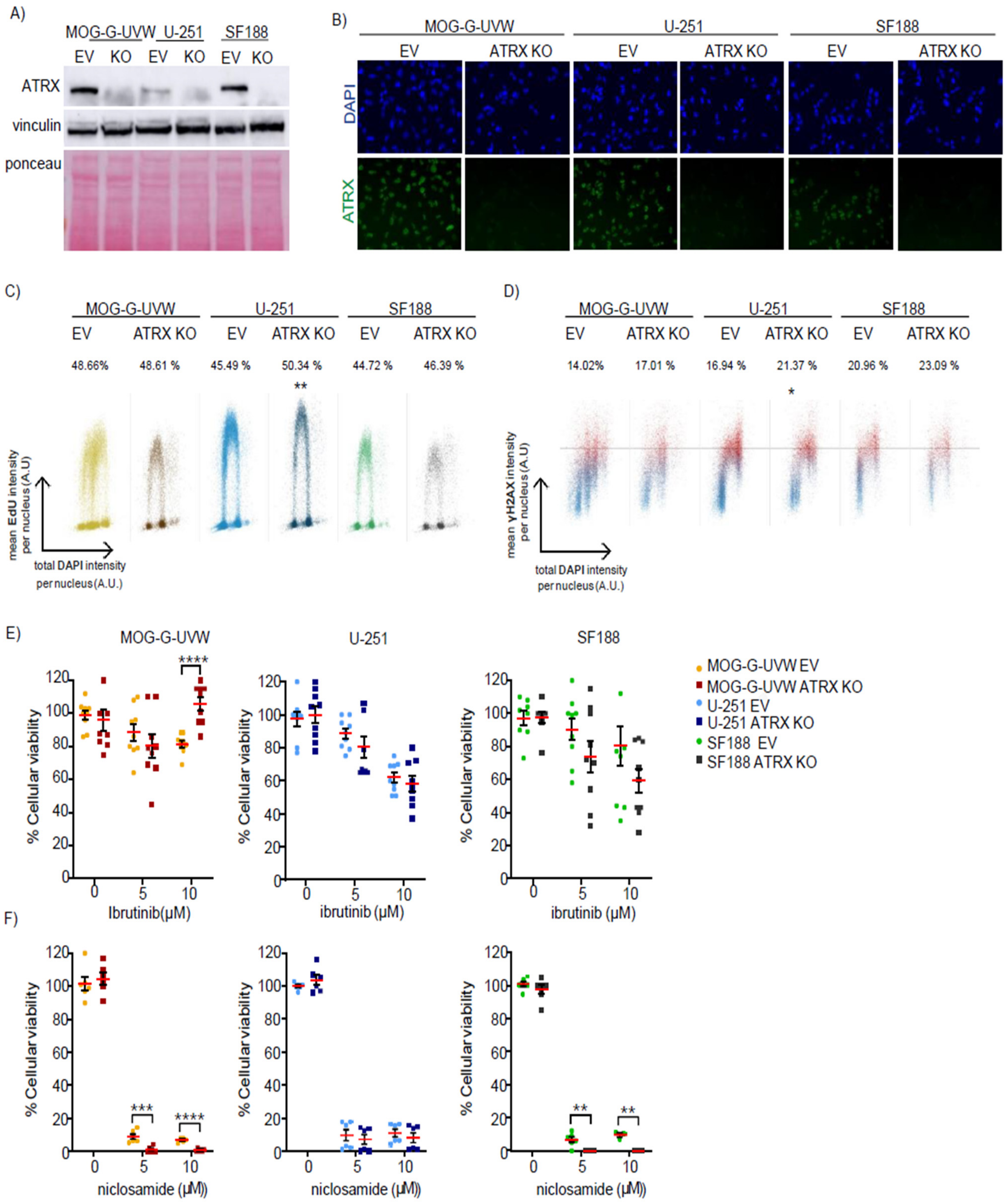

Figure S3. CRISPR/Cas9 engineered MOG-G-UVW, U-251 and SF188 (EV and ATRX KO) high-grade glioma cells: ATRX expression, effect on EdU incorporation and γH2AX levels, and sensitivity to BTK and STAT3 inhibitors. (**A**) Immunoblotting of MOG-G-UVW, U-251 and SF188 (EV and ATRX KO) cells with the indicated antibodies. (**B**) Assessment of ATRX status of MOG-G-UVW, U-251 and SF188 (EV and ATRX KO) cells by IF. (**C**)QIBC of MOG-G-UVW, U-251 and SF188 was performed and total DAPI/mean EdU intensities were plotted in scatter diagrams showing replication profiles. Percentages of EdU positive cells and statistics for significant conditions are shown. ATRX KO cells were compared to their ATRX WT counterparts. Significance was assessed by unpaired t test **$p \leq 0.01$. (**D**) QIBC of immunolabeled MOG-G-UVW, U-251 and SF188 was performed and total DAPI/mean γH2AX intensities were plotted in scatter diagrams. Red dots indicate γH2AX positive cells. Percentages of γH2AXpositive cells and statistics for significant conditions

are shown. ATRX KO cells were compared to their ATRX WT counterparts. Significance was assessed by unpaired t test *$p \leq 0.05$. (**E,F**) Cellular viability of MOG-G-UVW, U-251 and SF-188 (EV and ATRX KO) after 48h of treatment with the indicated drugs and concentrations compared to DMSO controls. Data shown corresponds to biological triplicates. Statistics for significant conditions means andSEMs are shown. Significance was assessed by unpaired t-test **$p \leq 0.01$; ***$p \leq 0.001$;****$p \leq 0.0001$.

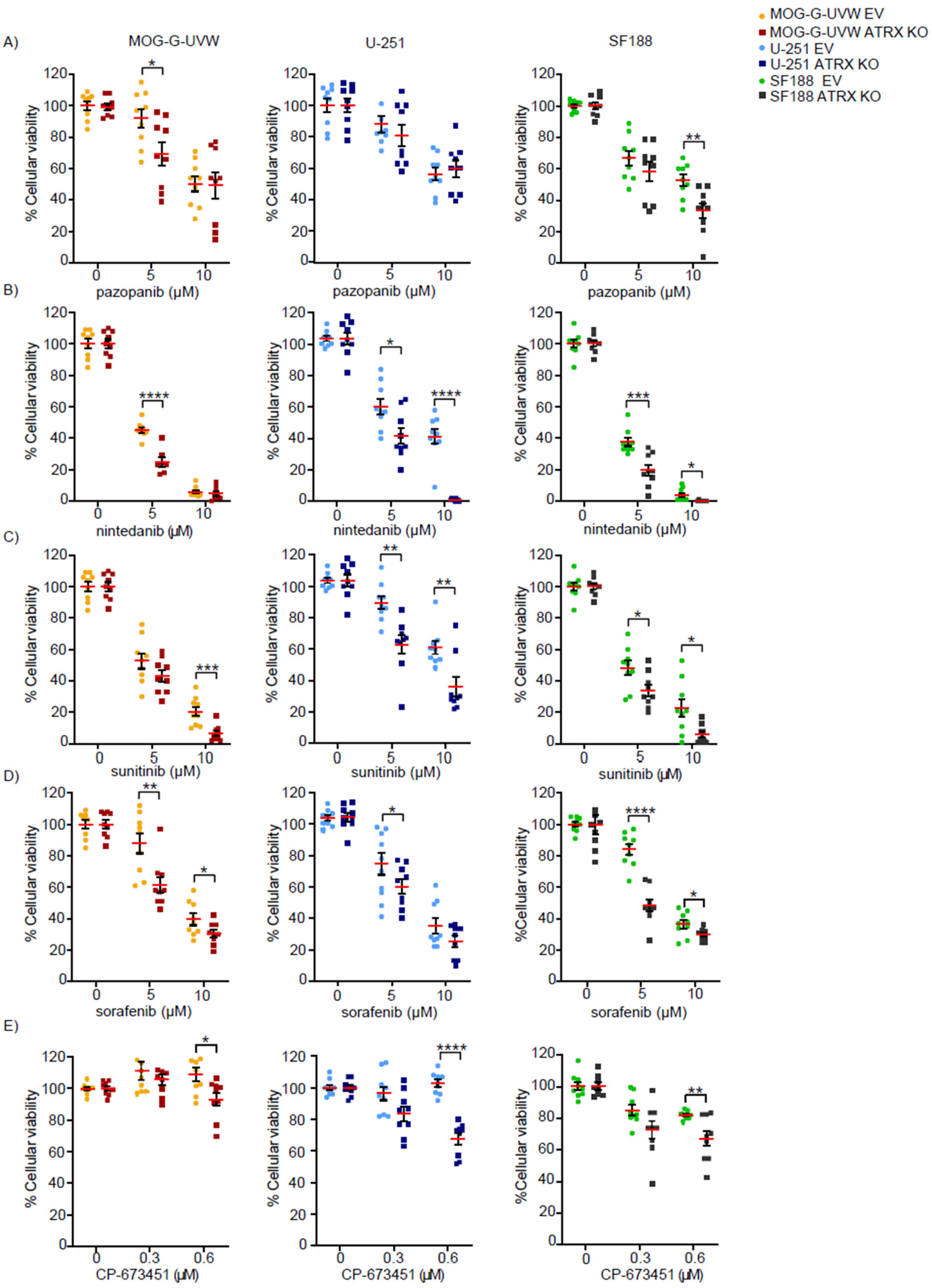

Figure S4. Cell viability of MOG-G-UVW, U-251 and SF-188 (EV and ATRX KO) upon treatment with RTK and PDGFR inhibitors. (**A–E**) Cellular viability of MOG-G-UVW, U 251 and SF 188 (EV and ATRX KO) after 48 h of treatment with the indicated drugs and concentrations compared to DMSO controls Data shown corresponds to biological triplicates. Statistics for significant conditions, means and SEMs are shown Significance was assessed by unpaired t test *p≤0.05; **p≤0.01; ***p≤0.001;****p≤0.0001.

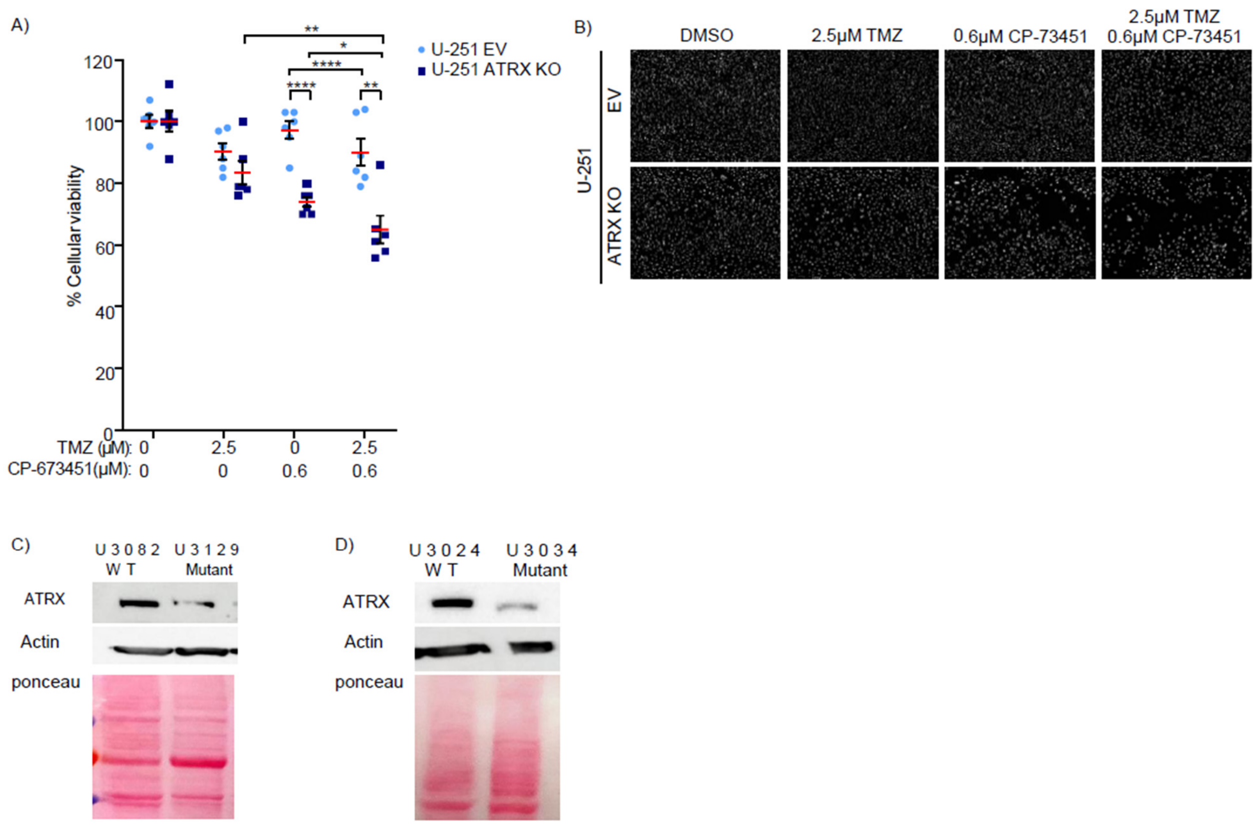

Figure S5. ATRX-deficient high-grade glioma cells are sensitive to combinatorial treatments of TMZ and CP-673451. (**A**) Cellular viability of U-251 (EV and ATRX KO) high-grade glioma cells after 48h of treatment with the indicated drugs and concentrations compared to DMSO controls. Data shown corresponds to two independent experiments. Mean and SEMs are indicated. Statistics for significant conditions are shown. Significance was assessed by unpaired t-test. *$p \leq 0.05$; **$p \leq 0.01$; ****$p \leq 0.0001$. (**B**) Representative images of U-251 (EV and ATRX-KO) high-grade glioma cells after 48h of treatment with either DMSO or the indicated drugs and concentrations. (**C**) Immunoblotting of U-3082 (ATRX WT) and U-3129 (ATRX mutant) cells with the indicated antibodies. (**D**) Immunoblotting of U-3024 (ATRX WT) and U-3034 (ATRX mutant) cells with the indicated antibodies.

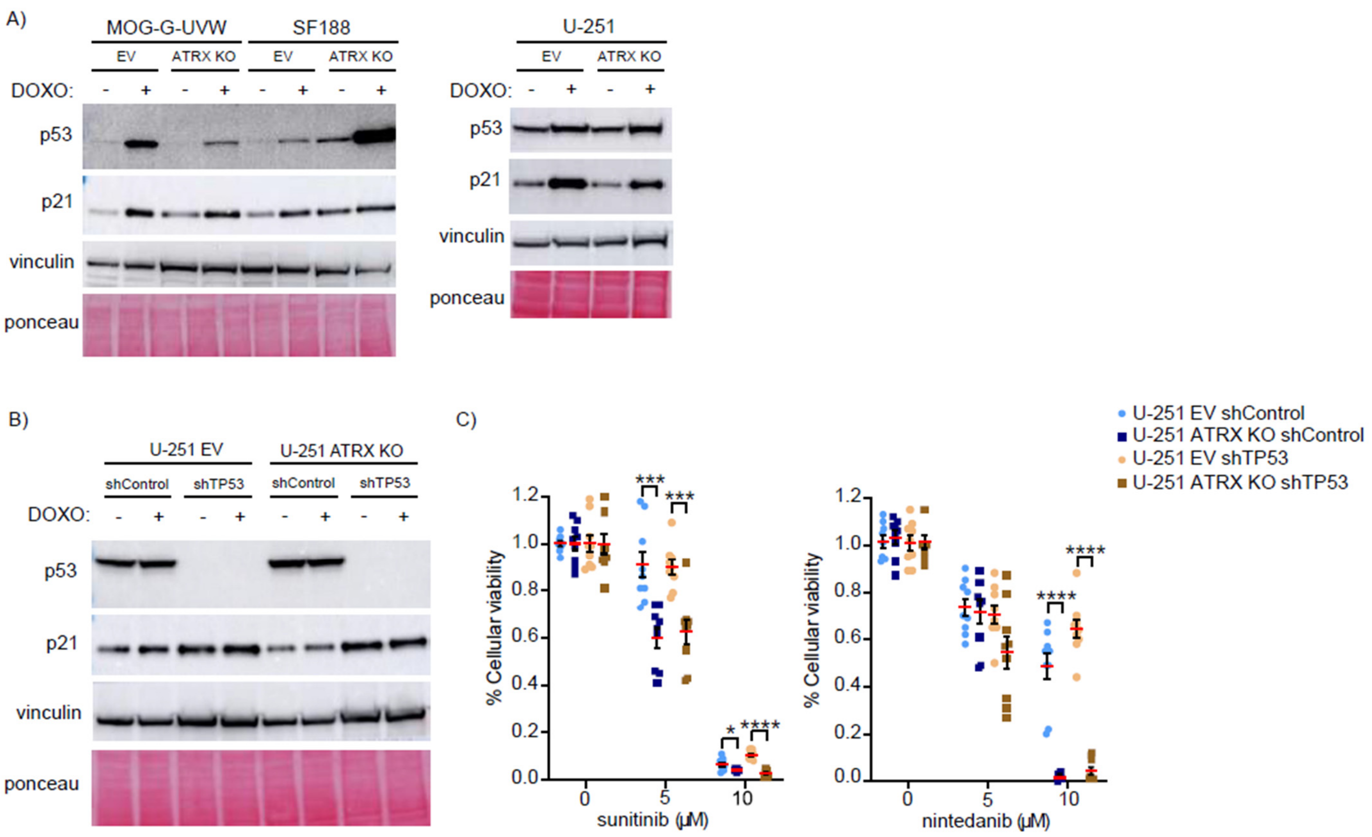

Figure S6. TP53 loss does not influence the toxicity of RTK inhibitors in ATRX-KO U-251 high-grade glioma cells. (**A**) Immunoblotting of MOG-G-UVW, SF188 and U-251 (EV and ATRX KO) cells with the indicated antibodies. Cells were untreated or treated with doxorubicin (0.5μM) for 12h. (**B**) Immunoblotting of U-251 (EV and ATRX-KO) cells infected with shControl or shTP53 with the indicated antibodies. Cells were untreated or treated with doxorubicin (0.5μM) for 12h. (**C**) Cellular viability of U-251 (EV and ATRX KO) infected with shControl or shTP53, after 48h of treatment with the indicated drugs and concentrations compared to DMSO controls. Data shown correspond to three independent experiments. Mean and SEMS are indicated. Statistics for significant conditions are shown. Significance was assessed by unpaired t-test. *$p \leq 0.05$; ***$p \leq 0.001$; ****$p \leq 0.0001$.
